# A distinct neuromelanin magnetic resonance imaging pattern in parkinsonian multiple system atrophy

**DOI:** 10.1186/s12883-020-02007-5

**Published:** 2020-11-27

**Authors:** Rita Moiron Simões, Ana Castro Caldas, Joana Grilo, Daisy Correia, Carla Guerreiro, Patrícia Pita Lobo, Anabela Valadas, Marguerita Fabbri, Leonor Correia Guedes, Miguel Coelho, Mario Miguel Rosa, Joaquim J. Ferreira, Sofia Reimão

**Affiliations:** 1 Neurology Department, Hospital Beatriz Ângelo, Loures, Portugal; 2 CNS-Campus Neurológico Sénior, Torres Vedras, Portugal; 3 Instituto de Medicina Molecular João Lobo Antunes, Faculdade de Medicina, Universidade de Lisboa, Av. Prof. Egas Moniz, 1649-028 Lisbon, Portugal; 4 Laboratório de Farmacologia Clínica e Terapêutica, Faculdade de Medicina, Universidade de Lisboa, Lisbon, Portugal; 5 Institute for Systems and Robotics (LARSyS), Department of Bioengineering, Instituto Superior Técnico, University of Lisbon, Lisbon, Portugal; 6 Department of Neurological Imaging, Hospital de Santa Maria, Centro Hospitalar Lisboa Norte, Lisbon, Portugal; 7 Imaging University Clinic, Faculdade de Medicina da Universidade de Lisboa, Lisbon, Portugal; 8 Department of Neurosciences and Mental Health, Serviço de Neurologia, Hospital de Santa Maria, Centro Hospitalar Lisboa Norte, Lisbon, Portugal; 9 Department of Neurosciences, clinical investigation center CIC 1436, Parkinson Toulouse expert center, NS-Park/FCRIN network and NeuroToul COEN center, Toulouse University Hospital, INSERM, University of Toulouse 3, Toulouse, France

**Keywords:** Multiple system atrophy, Neuromelanin, Susceptibility-weighted imaging, Nigrosome 1, MRI

## Abstract

**Background:**

Parkinsonian variant of multiple system atrophy is a neurodegenerative disorder frequently misdiagnosed as Parkinson’s disease. No early imaging biomarkers currently differentiate these disorders.

**Methods:**

Simple visual imaging analysis of the *substantia nigra* and *locus coeruleus* in neuromelanin-sensitive magnetic resonance imaging and nigrosome 1 in susceptibility-weighted sequences was performed in thirty patients with parkinsonian variant of multiple system atrophy fulfilling possible/probable second consensus diagnostic criteria. The neuromelanin visual pattern was compared to patients with Parkinson’s disease with the same disease duration (*n* = 10) and healthy controls (*n* = 10). *Substantia nigra* semi-automated neuromelanin area/signal intensity was compared to the visual data.

**Results:**

Groups were similar in age, sex, disease duration, and levodopa equivalent dose. Hoehn & Yahr stage was higher in parkinsonian multiple system atrophy patients, 69% of whom had normal neuromelanin size/signal, significantly different from Parkinson’s disease patients, and similar to controls. Nigrosome 1 signal was lost in 74% of parkinsonian multiple system atrophy patients. Semi-automated neuromelanin *substantia nigra* signal, but not area, measurements were able to differentiate groups.

**Conclusions:**

In patients with parkinsonism, simple visual magnetic resonance imaging analysis showing normal neuromelanin *substantia nigra* and *locus coeruleus,* combined with nigrosome 1 loss, allowed the distinction of the parkinsonian variant of multiple system atrophy from Parkinson’s disease and healthy controls. This easy and widely available method was superior to semi-automated measurements in identifying specific imaging changes in *substantia nigra* and *locus coeruleus*.

**Supplementary Information:**

The online version contains supplementary material available at 10.1186/s12883-020-02007-5.

## Background

Multiple system atrophy (MSA) is a sporadic, adult-onset and rapidly progressive neurodegenerative disorder, involving striatonigral, olivopontocerebellar, pyramidal, and autonomic systems. Clinical phenotypes are classified according to the most prominent motor symptom as parkinsonian (MSA-P) or cerebellar (MSA-C) [1].

The parkinsonian variant (MSA-P) has significant clinical overlap with Parkinson’s disease (PD), leading to frequent misdiagnosis [2], especially in the early stages, when red flags may be absent [3]. A *post mortem* study of neuropathologically-confirmed MSA-P showed that most patients were initially clinically diagnosed as PD and in half, the diagnosis was later changed to MSA [2].

Although less prevalent than PD, this devastating disorder is an important differential diagnosis to consider when evaluating a patient presenting with parkinsonism as there are significant prognostic and therapeutic implications.

*Substantia nigra* (SN) and *locus coeruleus* (LC) are dopaminergic and noradrenergic regions known to be selectively affected in PD and MSA-P [4, 5]. New magnetic resonance imaging (MRI) sequences have been recently developed, allowing the visualization of SN and LC in vivo and correlating with neuronal loss and iron deposition, two hallmarks of neurodegeneration [6–17].

Specific T1-weighted images show the SN and the LC as high-intensity signal regions due to the paramagnetic properties of neuromelanin (NM), a by-product of dopamine and noradrenaline metabolism that physiologically accumulates in neurons [6]. A specific pattern of NM loss has been described in PD patients, with both quantitative [7–9] and qualitative [10–12] methods. Few studies have used NM-MRI in MSA-P patients and the findings were inconclusive [18–20].

Iron content can be studied with MRI-specific sequences such as susceptibility-weighted imaging (SWI). A poor iron-binding region in the dorsolateral SN, nigrosome 1 (N1), is visualized as a hyperintense signal resembling a “swallow tail” in healthy individuals and its loss has been shown in PD and other degenerative parkinsonisms [13–17], including in MSA [17].

Accurate and easy to use diagnostic tools that can differentiate PD from MSA-P in early disease stages are greatly needed in clinical practice.

This study aimed to analyze the NM imaging visual pattern of the SN and LC in combination with the SWI N1 signal in patients with MSA-P. To the best of our knowledge, this is the first study in MSA-P patients combining visual analysis of NM and N1 SWI images.

## Methods

### Study population and historical controls

MSA patients from Campus Neurológico Sénior were identified from a movement disorders imaging database and selected if a 3 T MRI with a devoted movement disorders predefined protocol [6, 7] was performed. Clinical characteristics were assessed by two movement disorders specialists (RMS, ACC): disease duration since onset of first symptom and since diagnosis, presenting symptom, presence of cerebellar signs, Hoehn and Yahr stage [HY], levodopa equivalent daily dose [LEDD]. Only patients with parkinsonism and fulfilling second consensus criteria for possible or probable MSA [1] were included. PD patients with 2 to 5 years of disease duration (PD2_5y), and healthy controls (HC), who were included in previous studies, were selected to match disease duration in our study population [7, 11, 21, 22]. The study was approved by the local Ethical Committee and complied with national legislation and the Declaration of Helsinki guidelines.

### Imaging protocol

A NM and SWI MR protocol was performed using a 3.0 T Phillips scanner (Philips Achieva; Philips Medical Systems, Best, The Netherlands). The NM-sensitive pulse sequence parameters were: T1-weighted fast spin-echo; repetition time/effective echo time 633/10 ms; echo train length 3; number of slices 20; slice thickness 2.5 mm; intersection gaps 0 mm; matrix size 548 × 474; field of view 220 mm (pixel size 0.40 × 0.40 mm); acquisition time 8 min, adapted from the previous description by Sasaki et al [6, 7]. Slices were set in an oblique axial plane perpendicular to the fourth ventricle floor and covering from the posterior commissure to the inferior border of the pons, as previously described [6, 7].

The axial SWI sequence parameters were: repetition time/effective echo time 14/20 ms, flip angle of 10, number of slices 200, thickness 1/0.99/1, plane matrix 512,200 × 181, field of view 220 mm. Phase images were automatically filtered after acquisition, according to an established algorithm [23].

3 T T1-weighted sagittal sequences, obtained using a standard protocol with parameters used routinely in clinical practice [24], were additionally performed for morphometric brainstem analysis.

### Image analysis

**Fig. 1:**
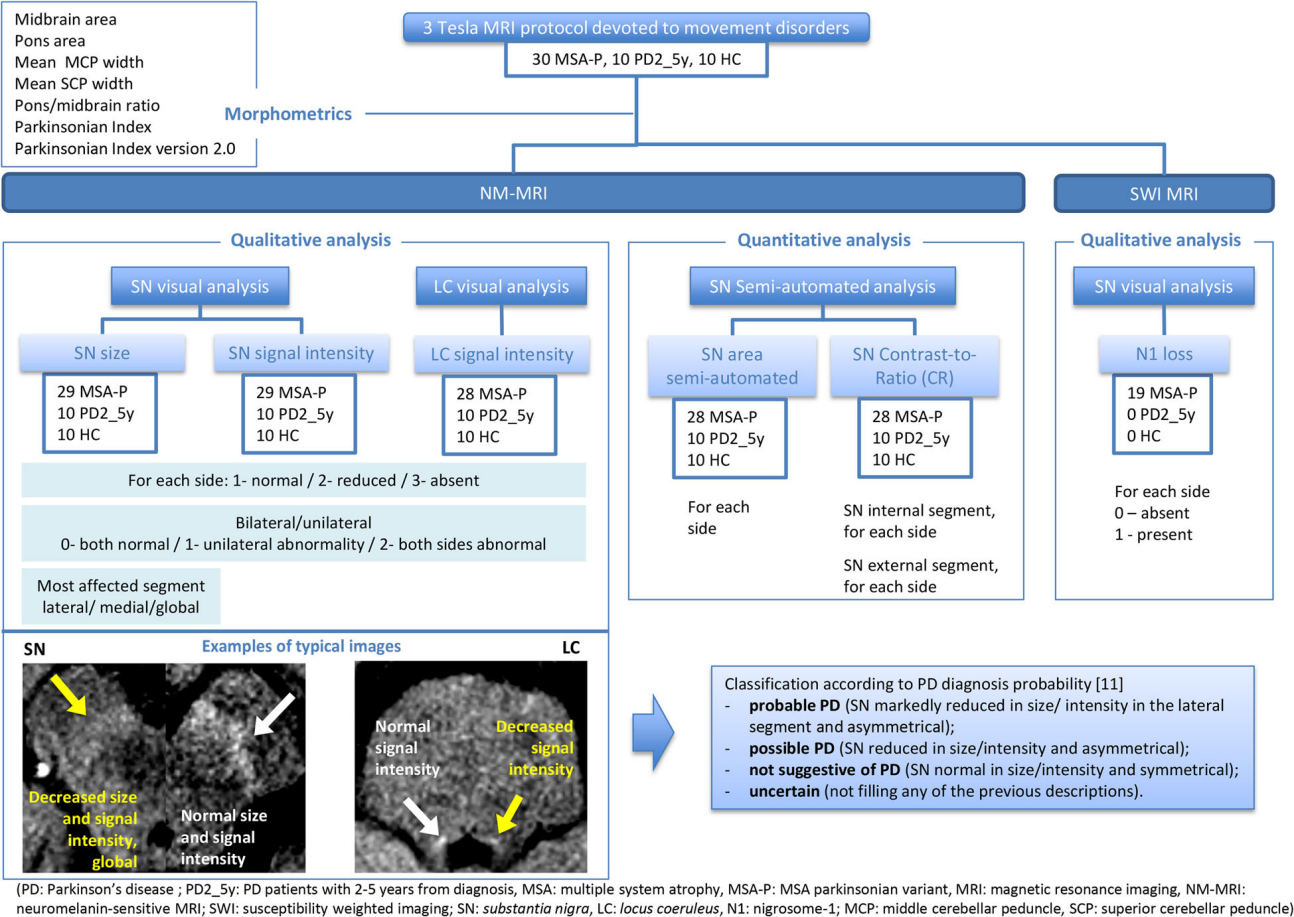
Imaging analysis and available sample size for each group and MRI modality

The following diagram (Fig. 1) summarizes the MRI imaging analyses that were performed***.***

#### Simple visual analysis

An experienced neuroradiologist (SR), blinded to the clinical diagnosis, performed a simple visual analysis of NM-sensitive MR images and SWI N1 signal.

Images were classified according to the presence of artifacts and their interference with the visual assessment of the NM high signal. Only the images that allowed a degree of confidence in evaluation that was equal to or higher than 50% were considered. Two MSA-P patients’ images had artifacts that interfered with the visual assessment of the NM high signal (one for both SN and LC, other for LC) (additional Table 1) and were excluded (degree of confidence in evaluation < 50%).

The SN size and signal intensity and the LC signal intensity in NM-MRI were classified using a three-point scale: 1–normal, 2–reduced, 3-absent. Both sides were rated independently. When applicable, the most affected segment was identified (1-lateral, 2-medial, 3-global) for each side.

Each participant was then classified as previously described [11] into one of four groups: probable PD (SN signal markedly reduced in size/ intensity in the lateral area and asymmetrical); possible PD (SN signal reduced in size/intensity and asymmetrical); not suggestive of PD (SN signal normal in size/intensity and symmetrical); or uncertain (not filling any of the previous descriptions).

The N1 image in SWI was categorized as 1-present or 2-absent for each side. SWI images were only available for MSA-P patients.

#### Morphometrics

The T1-weighted sagittal 3 T images were used to calculate brainstem metrics (midbrain area, pons area, middle cerebellar peduncle width and superior cerebellar peduncle width, III ventricle width, frontal horn width) using previously described methods [25, 26]. Pons/midbrain ratio, parkinsonian index and parkinsonian index version 2.0 were also calculated [25, 26].

#### Semi-automated imaging analysis

SN NM-sensitive images were additionally analyzed using semi-automated measurements of the area and signal intensity [contrast-to-ratio (CR) of SN internal and external segments], as previously described [6, 7, 11, 21, 22].

### Statistical analysis

The X^2^ test was used to compare the visual analysis results and ANOVA was used to compare the quantitative measurements in the three groups. For quantitative measurements with equal variances assumed, Bonferroni multiple comparisons post hoc analysis were performed to compare between the three groups. The *t-*test for independent samples was used to compare findings in MSA-P and PD2_5y.

To compare the visual analysis results of SN size and signal intensity with the semi-automated area and CR measurements, each individual SN was considered. In order to account for left/right differences in SN area and CR measurements, an asymmetry index (lower value/higher value) was calculated for area, CR internal segment and CR external segment for each subject. Differences in quantitative measurements between groups were evaluated by ANOVA test with post hoc Bonferroni correction.

Sensitivity, specificity, accuracy, negative predictive value (NPV), and positive predictive value (PPV) of visual analysis of NM-MRI for distinguishing PD2_5y from non-PD (MSA-P and HC) were calculated using a contingency table. “Probable PD” or “Possible PD” classifications were merged into the classification “PD”. Classifications of “uncertain” were considered false positives when attributed to MSA-P or HC and false negatives when attributed to PD2_5y.

Discriminative ability of quantitative methods of SN NM-MRI was evaluated by calculating the Receiver Operating Characteristic (ROC) curve and the area under the curve (AUC) for each group and each quantitative analysis.

A *p*-value of 0.05 was considered significant.

All analyses were performed with the IBM SPSS software version 24.

## Results

### Demographic and clinical characteristics of MSA-P, PD2_5y, and HC

The study included 30 patients with MSA-P, 10 PD2_5y and 10 HC. Table 1 shows the demographic and clinical characteristics of the participants at the time of MRI.

**Table 1:** Demographic and clinical characteristics of patients with MSA-P, PD 2–5 years duration and healthy controls

	MSA-P	PD 2–5 years	Healthy controls	p
N	30	10	10	
**DEMOGRAPHIC AND CLINICAL CHARACTERISTICS**				
**Sex – males (%)**	12 (40%)	8 (80%)	6 (60%)	0.077
**Age at MRI (years)**	66.8 ± 8.4	66.9 ± 6,1	61.4 ± 7.0	0.161
**MSA 2**^**nd**^ **consensus criteria**		NA	NA	NA
**- Possible MSA**	22 (73%)			
**- Probable MSA**	8 (27%)			
**Time from 1**^**st**^ **symptom at MRI, mean ± SD/median (years)**	4.6 ± 2.7 /4 (2 missing values)	NA	NA	NA
**Time from diagnosis at MRI, mean ± SD /median (years)**	2.4 ± 2.7 /1.2 (2 missing values)	NA	NA	NA
**Presenting symptom**		NA	NA	NA
**- motor-parkinsonism**	23/28 (82%)			
**- dysautonomia**	4/28 (14%)			
**- other**	1/28 (4%) (2 missing values)			
**Presence of cerebellar signs**	8/27 (30%) (3 missing values)	NA	NA	
**Hoehn & Yahr stage at MRI, mean/median**	**2.5 ± 0.5/2** (5 missing values)	**2,0 ± 0,0/2** (2 missing values)	NA	**< 0.001**
**LEDD at MRI (mg/day)**	674 ± 483 (4 missing values)	562 ± 279 (1 missing values)	NA	0.527

Bold values mean significant statistical differences

*Abbreviations*: *MSA-P*: Multiple system atrophy parkinsonian variant, *PD* Parkinson disease, *MRI* Magnetic resonance imaging, *NA* Not applicable, *SD* Standard Deviation, *LEDD* Levodopa equivalent daily dose

There were no differences in sex or age distribution between the groups (*p* = 0.077, *p* = 0.161, respectively). MSA-P and PD patients had the same disease duration. LEDD was not different but HY stage was higher in MSA-P (2.5 ± 0.5 vs. 2.0 ± 0.0, *p* < 0.001).

### Simple visual analysis of SN and LC in MSA-P patients

#### Neuromelanin visual analysis

*SN size* was normal in 69% (20/29). Of those with reduced SN size, the reduction was symmetrical in 5/9 and the lateral SN segment was the most affected (7/9) (Fig. 1.1, additional Table 1). The *SN signal intensity* was normal in 69% (20/29) (Fig. 2.2, Fig. 3a). None had absent SN signal. The proportion of patients with criteria for possible vs. probable MSA was similar in those with reduced SN size/signal intensity and those with normal SN (*p* = 0.872/*p* = 0.438).

**Fig. 2:**
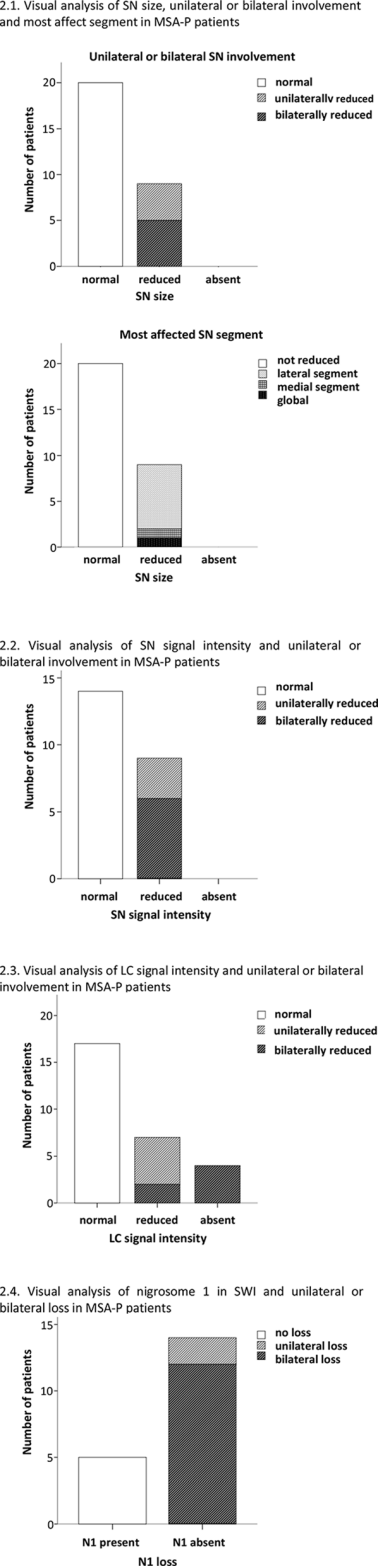
Visual analysis of SN and LC in NM-MRI and nigrosome-1 in SWI in patients with MSA-P

**Fig. 3:**
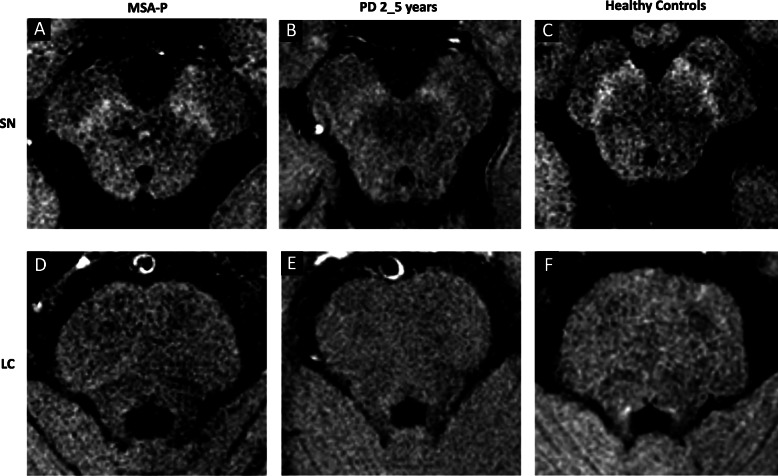
Neuromelanin-sensitive MRI axial images of SN (**a**-**c**) and LC (**d**-**f**) in a patient with MSA-P (**a**, **d**), PD with 2–5 years of duration (**b**, **e**) and a healthy control (**c**, **f**)

LC signal intensity was normal in 61% (17/28). In patients with reduced/absent LC signal, the reduction was bilateral in 54% (6/11) (Fig. 2.3, Fig. 3d, additional Table 1).

Thirteen patients (43%) had normal visual analysis of both SN and LC; five patients (17%) had abnormal SN and LC (size or signal intensity); six patients (20%) had normal SN size/signal intensity but abnormal LC, and four patients (13%) had abnormal SN size/signal intensity and normal LC. Of the eight patients classified as probable MSA-P, visual analysis showed that none had abnormal LC and four had both normal SN and LC. The HY stage tended to be more severe (2.8 ± 0.6) in patients with both normal SN and LC NM visual analysis and less severe in those with abnormal analysis of both structures (2.0 ± 0.0) (*p* = 0.081). These subgroups were not different in age (*p* = 0.580), time from first symptom (*p* = 0.453), time from diagnosis (*p* = 0.467) or LEDD (*p* = 0.240).

#### Nigrosome-1 visual analysis

N1 visual analysis of MSA-P patients is shown in Fig. 2.4 and additional Table 1. Patients with N1 loss (14/19) and without N1 loss (5/19) were not different in SN size, SN signal intensity or LC signal intensity (*p* = 0–637, *p* = 0.709, *p* = 0.459). Of the five MSA-P patients with N1 signal, two had normal NM visual analysis.

Disease duration since diagnosis, HY stage and LEDD, were similar in patients with N1 and those with N1 loss (*p* = 0.610, *p* = 0.608, *p* = 0.804, *p* = 0.725, respectively).

### Comparison of visual analysis in the three groups

#### Comparison of neuromelanin visual analysis in MSA-P, PD2_5y, and HC

**Fig. 4:**
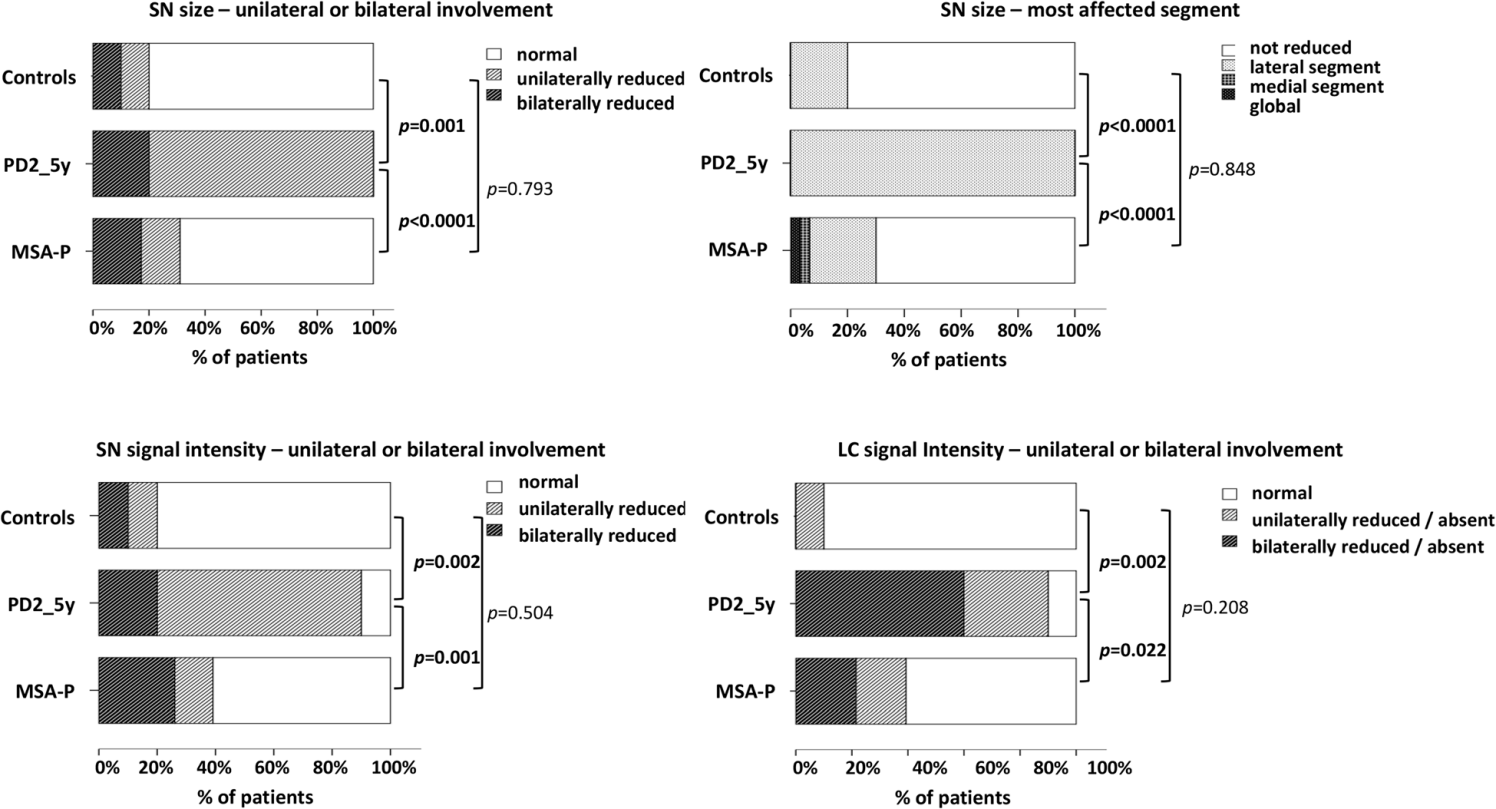
Comparison of the visual pattern of SN size, SN signal intensity and LC signal intensity in NM-MRI, in MSA-P, PD 2–5 years of duration and healthy controls

Additional Table 1 presents the results of visual analysis in PD2_5y and HC groups. The visual pattern of SN and LC in NM-MRI was different in MSA-P vs. PD2_5y and in PD2_5y vs. HC, but was similar in MSA-P and HC (Fig. 3, Fig. 4).

#### Ability of visual analysis of NM-MRI to discriminate between PD and non-PD

**Fig. 5:**
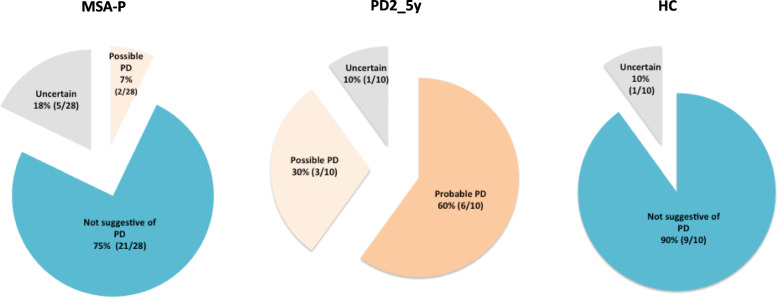
Classification of NM-MRI visual SN pattern in each group according to the radiological diagnosis of PD (probable PD, possible PD, not suggestive of PD and uncertain)

The SN NM-MRI visual pattern was classified as suggestive of PD (probable or possible) vs. not suggestive of PD vs. uncertain, for each subject (Fig. 5).

The visual pattern of SN in NM-MRI suggesting PD, had a sensitivity of 90%, specificity of 81%, accuracy of 83%, NPV of 98%, and PPV of 50% for the diagnosis of PD and to distinguish PD from MSA and HC.

### Morphometrics

Middle cerebellar peduncle width in sagittal T1, pons/midbrain ratio, parkinsonian index and parkinsonism index version 2.0 were able to discriminate MSA-P from PD2_5y and HC, but not PD2_5y from HC (additional Table 2).

### Semi-automated imaging analysis

Additional Table 1 shows the semi-automated measurements in the three groups.

Comparing the visual rates of SN size with the SN area measured by semi-automated methods, for each individual SN, no differences were found between those considered having normal size vs. reduced size by visual inspection (28.7 ± 10.8 vs. 26.2 ± 10.2, *p* = 0.333). CR in the internal SN, for each individual SN, tended to be lower in participants who had reduced SN intensity signal by visual inspection (1.1 ± 0.1 vs. 1.2 ± 0.0, *p* = 0.052); but CR in the lateral SN was similar in participants with normal and abnormal SN signal visual inspection (1.1 ± 0.0 vs. 1.1 ± 0.1, *p* = 0.185).

### Comparison of quantitative analysis in the three groups

#### Comparison of quantitative analysis of SN NM in MSA-P, PD2_5y and HC

SN semi-automated area was statistically different between the diseased groups and HC, but did not differ between PD2_5y and MSA-P (additional Table 1).

In contrast, mean CR of the internal and external SN segments were different in PD2_5y and MSA-P (additional Table 1). But mean CR of the SN internal segment did not distinguish PD2_5y or MSA-P from HC. Mean CR of the SN external segment was also similar in HC and MSA-P (additional Table 1). Left and right SN values for each parameter, measured by the asymmetry index, did not differ between groups (additional Table 1).

#### Ability of quantitative analysis of SN NM-MRI to discriminate between PD and non-PD

**Fig. 6:**
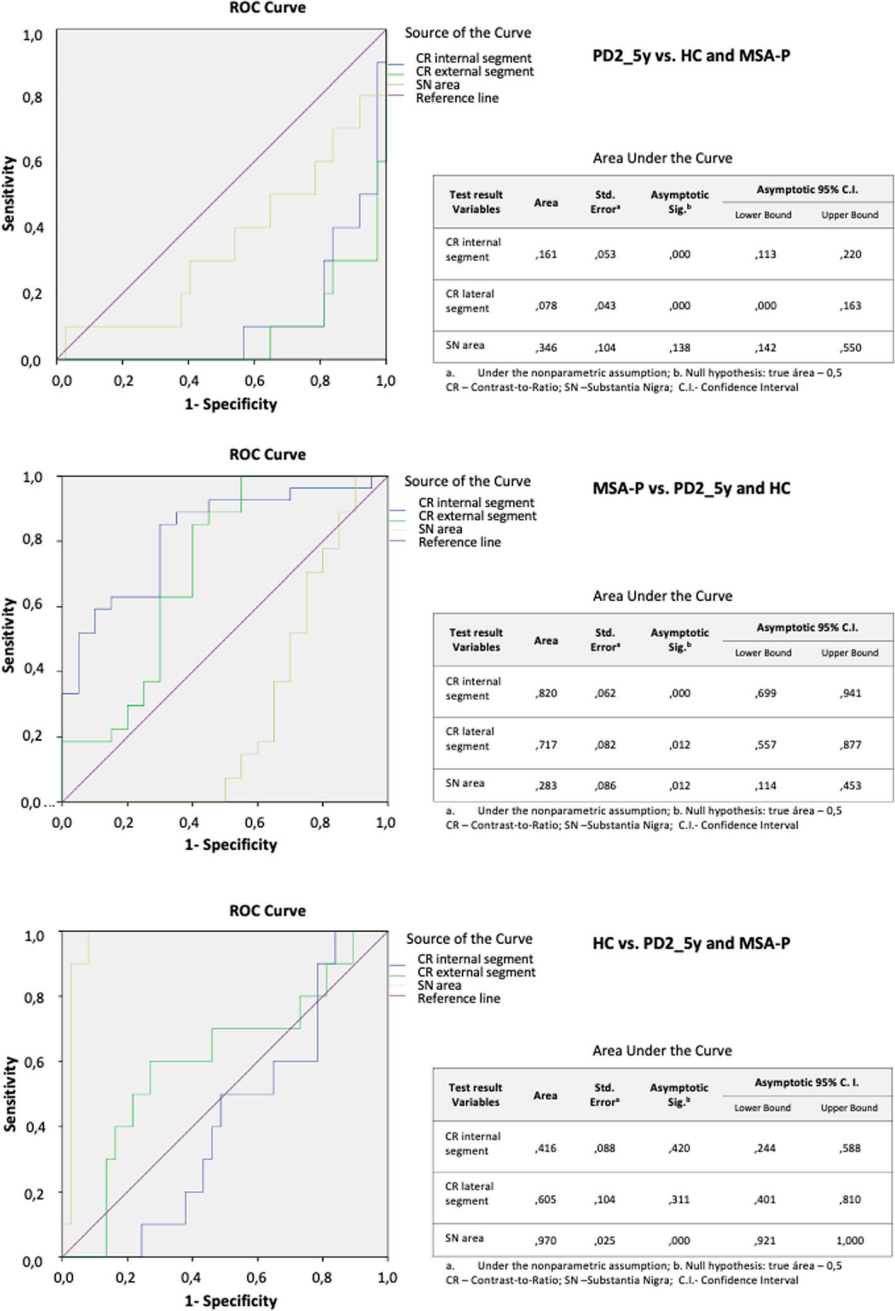
Ability of quantitative methods to discriminate between MSA-P, PD2_5y and HC, evaluated by ROC curves

Figure 6 shows the ROC curves and AUC considering SN mean area and mean CR of both lateral and internal segments to discriminate each group from the others. None of these parameters had good discriminatory ability for distinguishing PD from MSA-P or HC.

CR of both lateral and internal SN segments seems to be able to discriminate MSA-P from HC and PD2_5y (AUC = 0.71, AUC = 0.82). The mean SN area discriminates HC from diseased groups with high sensitivity and specificity (AUC = 0.97).

## Discussion

This is the first study to use simple visual analysis of NM-MRI in patients with MSA-P. Most MSA-P patients diagnosed according to the gold standard second consensus clinical criteria [1], had normal SN size and signal intensity, and normal LC signal. This pattern was clearly distinct from the findings in PD patients with similar disease duration, and overlapped with the healthy controls. Loss of nigrosome-1 in SWI sequences, a pathological hallmark of neurodegeneration, may help to distinguish MSA-P from healthy controls.

SN and LC NM-MRI have been extensively studied in patients with PD, mostly using quantitative measurements (volume, area, width, CR) [8–11]. Qualitative visual analysis of NM SN and LC is an easy and fast imaging evaluation method, without the need for post-processing software, and was recently shown to have similar diagnostic accuracy to more time consuming quantitative methods [10–12]. In our study, and in agreement with previous studies using qualitative visual analysis, the majority of PD patients had a unilateral decrease of the SN size and signal intensity (90–100%), mostly affecting the lateral region, and a bilateral reduction of LC signal (90%) [6–12, 21].

None of the previous studies analyzing NM-MRI in MSA-P [18–20] used visual analysis of the SN or LC. Two studies measured the SN CR [18, 19] and one used the SN volume [20]. In these studies, the results did not differentiate PD from MSA, but the SN CR and SN volume were smaller in MSA than in controls [18, 20]. This contrasts with our findings in that the NM CR on the lateral and internal SN segments discriminated between MSA-P and PD. Additionally, the quantitative measurements of the SN signal intensity in our study mirror and support the findings of NM visual inspection of the SN.

We found a normal LC signal in 59% of MSA patients. When considering only MSA-P patients fulfilling criteria for probable MSA, all had normal LC signal intensity, suggesting a higher specificity of this finding. Previous findings related to LC CR were contradictory [18, 20]. Small (9 and 10 patients) [18, 19] and/or heterogeneous MSA samples (different MSA subtypes included) [19, 20] in previous studies may explain these discrepancies. A volumetric study that included a larger group of 28 MSA patients who had similar disease duration to our patient group (3.9 ± 2 years) was unable to find significant differences between PD and MSA [19]. The data presented by this group is not clear when it comes to MSA clinical subtype and since it is a Japanese study, where there is a higher prevalence of MSA-C [27], this group is probably not comparable to ours which specifically excludes MSA-C patients.

The most frequent MSA-P NM visual pattern in our study overlapped with that of normal controls. Although the loss of SN NM has been considered a biomarker of nigral degeneration in PD [6], most of our MSA-P patients had normal SN NM by visual inspection, despite a well-known presynaptic dopaminergic dysfunction.

*Postmortem* NM-MRI studies have shown that SN NM is directly related to the number of NM-containing neurons [28] and that degenerated neurons may still contain NM granules so that SN NM measurements may not reflect dopaminergic function [29]. This may explain the apparent dissociation between dopamine transporter imaging, which measures dopaminergic function [30] or dopamine levels [31], and NM-MRI in MSA-P.

In our MSA-P patients, the clue to nigral pathology was the loss of N1, usually bilaterally, which was described in 74%. Although SWI sequences were not available for analysis in HC, loss of N1 has been previously described as pathological and a hallmark of nigral degeneration [17]. Our results are supported by those reported by Reiter et al. who included a substantial number of MSA patients (*n* = 22) and found N1 loss in SWI 3 T MRI in all, being able to discriminate between MSA and HC with a 100% sensitivity and 97% specificity [17]. In this same study, loss of the “swallow-tail-sign” helped to diagnose degenerative parkinsonism, regardless of nosological entity, versus HC, with a sensitivity of 94% and specificity of 90% [17]. In our study, N1 loss analysis was not available for PD or HC. However, previous results strongly suggest that N1 loss is pathological and that healthy subjects have a preserved N1. Extrapolating this knowledge to our HC sample, we would expect that, although the NM pattern would be similar (not decreased in size or signal intensity) in both MSA-P and HC, the N1 loss in diseased subjects would help to set apart these two groups only based in MRI visual analysis.

Nigral degeneration in MSA-P is corroborated in our study by N1 loss, but it occurs without significant NM loss, by visual analysis. This contrasts to what has been described for PD in which there is both SN NM reduction (as also corroborated in our study) and N1 loss. Reimão et al. (2016) have shown that there is no technical interference of iron paramagnetic properties in the SN NM MRI signal, and the same study also suggested that NM loss and N1 loss in PD were independent mechanisms [32]. Our findings in MSA-P, dissociating NM and N1 signals, also support the hypothesis that iron deposition and NM loss may be distinct and independent pathophysiological mechanisms.

Also, the distinct NM and N1 signals in MSA-P and PD, may suggest that nigral dysfunction in these disorders would result from different mechanisms. Both are synucleinopathies, but α-synuclein aggregates have a distinct distribution, mostly affecting the dopaminergic neurons in PD and the olygodendrocytes in MSA. From our findings, one could expect that dopaminergic neuronal loss would be less pronounced in MSA than in PD and/or that remaining dopaminergic neurons in MSA would lose less NM than those in PD. *Post mortem* neuropathological studies in MSA do not corroborate our first hypothesis as severe duration-dependent neuronal loss in SN is described [2]. However, in these *post mortem* samples, few early-stage MSA patients were included (median disease duration of 7.3 years) [2, 5]. Supporting the second hypothesis, α-synuclein has been shown to inhibit melanin synthesis in dopaminergic neurons, lowering intracellular melanin content [33]. As neuronal α-synuclein inclusions predominate in PD, one may hypothesize that NM loss would be accelerated and consequently more severe in PD than MSA-P, for the same disease stage. This could justify the preserved visual inspection of SN and LC NM in most of our early patients in the present study. Decreasing SN signal and area with disease progression and severity has been described in PD [22, 34], and may also occur in MSA-P. However, in our study, a third of MSA-P had decreased SN size/signal intensity, mostly bilateral, but this subgroup was not clinically different from those with normal SN, therefore distinct disease stages do not explain these findings. Studies on MSA-P patients in different disease stages would be necessary to confirm this hypothesis.

Visual inspection of NM-MRI sequences seems to be more reliable to differentiate PD from MSA-P than quantitative measurements. In our study, visual inspection of NM in what relates to SN size, signal intensity and asymmetry can distinguish PD from MSA and HC with a sensitivity of 90% and a specificity of 80%. Quantitative SN area and CR of lateral and medial SN segments were not able to discriminate PD from MSA and HC. The AUC suggests that the mean SN area is able to discriminate healthy subjects from diseased patients with high sensitivity and specificity and the CR may distinguish MSA from healthy subjects and PD with moderate sensitivity and specificity.

However, although lateral and medial SN CRs were statistically different in MSA and PD, the time consuming method and the unavailability of a cut-off for this discrimination needs to be addressed in the future to allow it’s usage in clinical practice. Also, sensitivity and specificity seem to be lower than when using visual inspection of SN NM-MRI.

The inability of quantitative semi-automated measurements to discriminate between PD and MSA may be explained by the technique. These methods with region growing algorithms use a seeding point in a given region of interest (ROI) and compare it with adjacent areas. When there is a diffuse reduction in NM T1 high intensity signal in the SN, when placing the ROI, there will be no significant difference between neighboring pixels, and the automated methods will not be able to calculate the SN area/signal nor detect specific pattern changes. By contrast, visual analysis is able to detect the specific pattern of asymmetrical size and signal intensity reduction typical of PD that is not present in most MSA-P patients, leading to higher discriminatory ability than quantitative methods.

The major strengths of our study are the large and homogenous sample of MSA-P patients, the devoted movement disorders MRI protocol that was used and the previous experience of our neuroradiologist in visual rating of SN and LC in NM-MRI. However, in our MSA-P sample, only a small subset fulfilled second consensus criteria for probable MSA. The short disease duration of our patients (median from first symptom of 4 years and median time from diagnosis of 1 year), may prevent a full-blown clinical picture, decreasing the level of certainty for diagnosis. Additionally, we cannot exclude misdiagnosis in our MSA sample. In our study, a third of MSA-P had decreased SN area/signal intensity and this was mostly bilateral. Of these, in only 2/9, the combined analysis was suggestive of possible PD. The MSA diagnostic criteria have been criticized for having suboptimal diagnostic accuracy [35]. The tendency for MSA-P patients with abnormal LC and/or SN pattern to have a lower HY stage could suggest that these patients may have been wrongly diagnosed as MSA-P and corresponded in fact to PD.

Additionally, historic controls were used for comparison with our MSA-P patients. Although we tried to control for age, disease duration and LEDD in both diseased groups, the HY stage was statistically different. The higher HY in MSA-P was somehow expected, as MSA-P is a more severe disorder with less benefit from dopaminergic medication. Both disease duration [34] and disease severity measured by the HY [22] have been previously reported to correlate with reduced SN neuromelanin in PD. However, conclusions cannot be dropped for HY stage effect in NM SN signal in MSA-P patients as it seems that in these patients NM has a distinct behavior from PD. In fact, in our study, most MSA-P patients have normal SN NM size and signal intensity by visual inspection besides a higher HY, which was the opposite one would expect by the NM behavior in PD. The effect of this difference in these two groups is unknown and was controlled during statistical analysis.

An additional weakness was the unavailability of the SWI sequences for historic controls for comparison with MSA-P patients.

Also, simple visual analysis is an observer-dependent analysis. However, this would be minimized by expert reading of the images. Having had individual raters to perform the visual analysis and comparing the inter-rater agreement would have supported the validity of our results. Despite not having this double rating in our study, our group has previously published a simple visual inspection of SN neuromelanin images, read by individual raters, in which Cohen’s Kappa Coefficient showed fair to moderate inter-rater agreement (0.39 for SN signal/size, 0.45 for subject classification), supporting the validity of our qualitative results [11].

Future studies of combined visual analysis of the NM and N1 loss in patients with parkinsonism, with larger samples of clinically homogenous subtypes, will improve our understanding of the neuroimaging characteristics of these conditions. It would also be of interest to evaluate MSA-C and adult-onset spinocerebellar ataxias NM and N1 imaging findings.

## Conclusions

Our findings suggest that simple visual inspection of NM in the SN and LC may easily distinguish PD from MSA-P patients, even in early stages. PD patients have a specific imaging pattern that contrasts with a normal NM pattern in the majority of MSA-P patients. In our study, the presence of decreased unilateral SN and LC NM pattern was strongly suggestive of PD and excluded the diagnosis of MSA-P with a sensitivity of 90%, specificity of 81%, and accuracy of 83%. Combined visual inspection of NM-MRI and N1 in SWI is suggested to set apart non-PD degenerative parkinsonism from healthy subjects.

Experienced neuroradiologists can use these not time consuming and widely available imaging techniques to greatly help clinical evaluation of individual patients with parkinsonism.

## Supplementary Information

**Additional file 1: Additional Table 1** Qualitative and quantitative analysis of SN and LC in NM-sensitive MRI and SWI.

**Additional file 2: Additional Table 2** Morphometrics in MSA-P, PD with 2 to 5 years duration and healthy controls. (PD: Parkinson’s disease, MSA: multiple system atrophy, MSA-P: MSA parkinsonian variant, MRI: magnetic resonance imaging, SN: *substantia nigra*, LC: *locus coeruleus*, MCP: middle cerebellar peduncle, SCP: superior cerebellar peduncle). ¥ - Mean value is presented, as differences between left and right measurements were not significant . Bold values mean significant statistical differences.

## Data Availability

The datasets used and/or analyzed during the current study are available from the corresponding author on reasonable request.

## Abbreviations

CR: Contrast-to-ratio
HC: Healthy controls
HY: Hoehn & Yahr stage
LC: *Locus Coeruleus*
LEDD: Levodopa equivalent daily dose
MCP: Middle cerebellar peduncle
MRI: Magnetic resonance imaging
MSA-C: Cerebellar variant of multiple system atrophy
MSA-P: Parkinsonian variant of multiple system atrophy
MSA: Multiple System Atrophy
N1: Nigrosome 1
NA: Not applicable
NM-MRI: Neuromelanin-sensitive magnetic resonance imaging
NM: Neuromelanin
NPV: Negative predictive value
PD: Parkinson’s disease
PD2_5y: Parkinson’s disease patients with 2 to 5 years of disease duration
PPV: Positive predictive value
SCP: Superior cerebellar peduncle
SD: Standard deviation
SN: *Substantia nigra*
SWI: Susceptibility-weighted imaging
