# Supplementary material for: A distinct neuromelanin magnetic resonance imaging pattern in parkinsonian multiple system atrophy
Source: BMC Neurol. 2020 Nov 27;20:432. doi: 10.1186/s12883-020-02007-5 (PMC7694430; doi:10.1186/s12883-020-02007-5)
Supplement: Supplementary file 1 — Additional file 1: Additional Table 1 Qualitative and quantitative analysis of SN and LC in NM-sensitive MRI and SWI. [file 12883_2020_2007_MOESM1_ESM.docx]

**Additional table 1** – Qualitative and quantitative analysis of SN and LC in NM-sensitive MRI and SWI

|  | **MSA-P** | **PD 2-5 years** | **Healthy controls** | **p** |
| --- | --- | --- | --- | --- |
| Patients considered for evaluation | **30/30** | **10/10** | **10/10** |  |
| **QUALITATIVE ANALYSIS – SIMPLE VISUAL ANALYSIS** | | | | |
| **SN and LC visual analysis, NM-sensitive MRI** | | | | |
| ***SN size***  - Observer’s degree of confidence (low/moderate/high)  - Number of patients considered for visual rating (N/total)  - Number with normal symmetrical size (%)  - Number with reduced SN size  - Number/total (%)  - Symmetrical : asymmetrical  - Segment reduced - lateral : medial : global ^&^ | 1/6/23  29/30  20/29 (69%)  9/29 (31%)  5 : 4  7 : 1 : 1 | 0/0/10  **10/10**  0/10 (0%)  **10/10 (100%)**  2 : 8  10 : 0 : 0 | 0/0/10  10/10  8/10 (80%)  2/10 (20%)  1 : 1  2 : 0 : 0 | **p<0.001**  **- MSA-P vs. PD2_5y: p<0.001**  - MSA-P vs. HC: p=0.504  **- PD2_5 vs. HC: p<0.001**  (p values for unilateral/bilateral involvement and most affected segment are shown in graph 1.1.) |
| ***SN signal intensity***  - Observer’s degree of confidence (low/moderate/high)  - Number of patients considered for visual rating  *-* Number with normal symmetrical signal intensity (%)  - Number with reduced SN signal intensity  - Number/total (%)  - Degree of reduction (reduced, very reduced, absent)  - Symmetrical : asymmetrical | 1/5/24  29/30  20/29 (69%)  9/29 (31%)  9:0:0  7 : 2 | 0/0/10  **10/10**  **1/10 (10%)**  **9/10 (90%)**  **9:0:0**  **2 : 7** | 0/2/8  10/10  8/10 (80%)  2/10 (20%)  2:0:0  1 : 1 | **p=0.002**  **- MSA-P vs. PD2_5y: p=0.001**  - MSA-P vs. HC: p=0.504  **- PD2_5 vs. HC: p=0.002** |
| - **LC signal intensity**   - Observer’s degree of confidence in visual rating  (low/moderate/high)  - Number of patients considered for visual rating (N/total)  - Number with normal signal intensity  - Number with reduced LC signal intensity or absent signal  - Number/total (%)  - Degree of reduction (reduced but visible : not visible)^#^  - Symmetrical : asymmetrical | 2/7/21  28/30  17/28 (61%)  11/28 (39%)  7 : 4  6 : 5 | 0/0/10  **10/10**  **1/10 (10%)**  **9/10 (90%)**  **6:3**  **7 : 2** | 0/1/9  10/10  9/10 (90%)  1/10 (10%)  1/0  0 : 1 | **p=0.008**  **- MSA-P vs. PD2_5y: p=0.022**  - MSA-P vs. HC: p=0.208  **- PD2_5 vs. HC: p=0.002** |
| **Nigrosome 1, SWI** | | | | |
| - Number of patients considered for visual rating (N/total) | 19 | -- | -- | **NA** |
| - Number with nigrosome 1 present  - Number with absent nigrosome 1  - Number/total (%)  - Symmetrical : asymmetrical | 5/19 (26%)  14/19 (74%)  12 : 2 |  |  |  |
| **QUANTITATIVE ANALYSIS – SEMI-AUTOMATED METHODS** | | | | |
| **SN semi-automated areas, NM-sensitive MRI** | | | | |
| - right SN area  - left SN area  - mean SN area ((right + left) /2) | 24.43 ± 7.01  24.13 ± 6.54  24.16 ± 5.17  (2 NA) | 22.72 ± 10.62  24.02 ± 11.01  23.37 ± 10.36 | 40.73 ± 8.61  42.42 ± 7.49  41,58 ± 6.02 | **p<0.001^£^**  MSA-P vs. PD2_5y: p=1.000  **MSA-P vs. HC: p<0.001**  **PD2_5y vs HC: p<0.001** |
| - SN area asymmetry index (lower value/higher value) | 0.81 ± 0.18 | 0.80 ± 0.14 | 0.81 ± 0.12 | p=0.962 |
| **SN signal intensity, NM-sensitive MRI** | | | | |
| - right SN – CR internal  - left SN – CR internal  - mean CR internal ((right + left)/2) | 1.18 ± 0.06  1.17 ± 0.05  1.18 ± 0.05 | 1.13 ± 0.04  1.10 ± 0.04  1,12 ± 0.03 | 1.16 ± 0.02  1.14 ± 0.04  1.15 ± 0.02 | **p=0.002^£^**  **MSA-P vs. PD2_5y: p=0.001**  MSA-P vs HC p=0.324  PD2_5y vs HC: p=0.242 |
| - CR internal - asymmetry index (lower value/higher value) | 0.97 ± 0.02 | 0.96 ± 0.02 | 0.97 ± 0.02 | p=0.412 |
| - right SN – CR lateral  - left SN – CR lateral  - mean CR lateral ((right + left)/2) | 1.11 ± 0.04  1.08 ± 0.05  1.09 ± 0.04 | 1.02 ± 0.03  1.04 ± 0.05  1.03 ± 0.03 | 1.08 ± 0.05  1.10 ± 0.04  1.09 ± 0.03 | **p<0.001^£^**  **MSA-P vs. PD2_5y: p<0.001^€^**  MSA-P vs HC p=1.000  **PD2_5y vs HC: p=0.004** |
| - CR lateral - asymmetry index (lower value/higher value) | 0.95 ± 0.03 | 0.96 ± 0.03 | 0.95 ± 0.03 | p=0.954 |
| (PD: Parkinson’s disease, MSA: multiple system atrophy, MSA-P: MSA parkinsonian variant, MRI: magnetic resonance imaging, NM: neuromelanin, SN: *substantia nigra*, LC: *locus coeruleus*,  SWI: susceptibility-weighted imaging; NA: not applicable, CR: contrast-to-noise ratio)  & - when the signal was bilaterally reduced, the segment affected was the same in both sides for the same patient  # - Non-visible LC were all bilateral in MSA-P cases, one was unilateral in PD cases.  £ - statistical results similar for right, left and mean values, so results for mean area are presented  € - significant only for right CR lateral and mean CR lateral  Bold values mean significant statistical differences | | | | |
