# Supplementary material for: A distinct neuromelanin magnetic resonance imaging pattern in parkinsonian multiple system atrophy
Source: BMC Neurol. 2020 Nov 27;20:432. doi: 10.1186/s12883-020-02007-5 (PMC7694430; doi:10.1186/s12883-020-02007-5)
Supplement: Supplementary file 2 — Additional file 2: Additional Table 2 Morphometrics in MSA-P, PD with 2 to 5 years duration and healthy controls. (PD: Parkinson’s disease, MSA: multiple system atrophy, MSA-P: MSA parkinsonian variant, MRI: magnetic resonance imaging, SN: substantia nigra, LC: locus coeruleus, MCP: middle cerebellar peduncle, SCP: superior cerebellar peduncle). ¥ - Mean value is presented, as differences between left and right measurements were not significant . Bold values mean significant statistical differences. [file 12883_2020_2007_MOESM2_ESM.docx]

**Additional table 2** – Morphometrics in MSA-P, PD with 2 to 5 years duration and healthy controls.

| **Morphometrics** | **MSA-P** | **PD 2-5 years** | **Healthy Controls** | **p** |
| --- | --- | --- | --- | --- |
| - Midbrain area, sagittal T1 (cm^2^) | **1.21 ± 0.18** | **1.20 ± 0.15** | **1.40 ± 0.19** | **p=0.021**  **MSA-P vs. PD2_5y: p=1.000**  **MSA-P vs. HC p=0.027**  **PD2_5y vs. HC: p=0.053** |
| - Pons area, sagittal T1 (cm^2^) | 5.05 ± 0.67 | 5.55 ± 0.69 | 5.38 ± 0.36 | 0.083 |
| - Mean MCP width, sagittal T1^¥^ (cm) | **0.83 ± 0.11** | **0.95 ± 0.07** | **0.95 ± 0.09** | **p=0.001**  **MSA-P vs. PD2_5y: p=0.009**  **MSA-P vs. HC p=0.006**  PD2_5y vs. HC: p=1.000 |
| - Mean SCP width, sagittal T1^¥^ (cm) | 0.42 ± 0.05 | 0.41 ± 0.03 | 0.43 ± 0.06 | p=0.728 |
| - Pons/midbrain ratio | **4.24 ± 0.78** | **10.85 ± 1.96** | **8.84 ± 1.91** | **p<0.001**  **MSA-P vs. PD2_5y: p<0.001**  **MSA-P vs. HC: p<0.001**  **PD2_5y vs. HC: p=0.008** |
| - Parkinsonian Index   (pons area/midbrain area x mean MCP/mean SCP) | **8.42 ± 1.82** | **4.73 ± 0.86** | **3.90 ± 0.48** | **<0.001**  **MSA-P vs. PD2_5y: p<0.001**  **MSA-P vs. HC p<0.001**  PD2_5y vs. HC: p=0.694 |
| - Parkinsonian Index version 2.0   (Parkinsonian Index x III ventricle width/frontal horn width ratio) | **1.66 ± 0.65**  (1 NA) | **0.94 ± 0.36** | **0.59 ± 0.17** | **p<0.001**  **MSA-P vs. PD2_5y: p=0.003**  **MSA-P vs. HC p<0.001**  PD2_5y vs. HC: p=0.469 |

(PD: Parkinson’s disease, MSA: multiple system atrophy, MSA-P: MSA parkinsonian variant, MRI: magnetic resonance imaging, SN: *substantia nigra*, LC: *locus coeruleus*, MCP: middle cerebellar peduncle, SCP: superior cerebellar peduncle)

¥ - Mean value is presented, as differences between left and right measurements were not significant

Bold values mean significant statistical differences
